# Solitaire Stentectomy Using a Stent-Retriever Technique in a Porcine Model

**DOI:** 10.1007/s00062-020-00906-1

**Published:** 2020-05-18

**Authors:** Andreas Simgen, Michael Kettner, Frida Juliane Webelsiep, Toshiki Tomori, Ruben Mühl-Benninghaus, Umut Yilmaz, Pervinder Bhogal, Matthias W. Laschke, Michael D. Menger, Wolfgang Reith, Philipp Dietrich

**Affiliations:** 1 Department of Neuroradiology, Saarland University Hospital, Kirrbergerstraße1, 66424 Homburg/Saar, Germany; 2 Institute for Clinical & Experimental Surgery, Saarland University Hospital, Homburg/Saar, Germany; 3 Department of Interventional Neuroradiology, The Royal London Hospital, Whitechapel Road, E1 1BB London, UK

**Keywords:** Animal model, Trevo, Solitaire, EmboTrap II Revascularization Device, 3D Revascularization Device

## Abstract

**Purpose:**

Mechanical thrombectomy using the Solitaire device has become a standard treatment of ischemic stroke due to large vessel occlusions. Inadvertent detachment is a feared complication, which is associated with poor clinical outcome. The aim of this experimental study was to assess in a porcine model the feasibility and effectiveness of rescuing detached Solitaire devices using different stent retrievers.

**Methods:**

Solitaire FR devices (4 × 15/20 mm and 6 × 20/30 mm) were placed in the axillary artery of pigs. By means of 3 different stent retrievers (Trevo ProVue; EmboTrap II revascularization device; 3D revascularization device) a total of 24 rescue maneuvers (8 per retriever) were performed by deploying the retrievers within the deployed Solitaire devices and trapping parts of the Solitaire within the microcatheter. Rescue rates, rescue time and complications were assessed.

**Results:**

Overall stentectomy of the Solitaire devices was successful in all cases (100%). Time of rescue was comparable using the applied stent retrievers (Trevo ProVue; EmboTrap II revascularization device; 3D revascularization device). Complications, such as entrapment of the Solitaire-retriever complex at the intermediate catheter, Solitaire migration, vasospasm, perforation, or dissection were not observed.

**Conclusion:**

Stentectomy of inadvertently detached Solitaire devices using different stent retrievers is a feasible and effective method. Rescue rates and times with the Trevo ProVue, EmboTrap II and 3D revascularization device were comparable.

## Introduction

Since 2015, mechanical thrombectomy with retrievable stents has become a standard treatment of ischemic stroke due to large vessel occlusion with very good clinical outcome [1]. The Solitaire AB/FR device (Medtronic, Irvine, CA, USA) was the first fully retrievable and self-expanding intracranial stent available [2]. Initially, this device was designed for stent-assisted treatment of intracranial aneurysms [3]. In 2008, one of the first cases was described in the literature where a Solitaire was used to retrieve an intra-arterial thrombus [4]. Ever since, the Solitaire device passed through several revisions (Solitaire 2 and Solitaire Platinum), leading to the current 2019 version Solitaire X and is probably the most widely used stent-retriever in the world. Incidences of device-related complications have been reported in literature between <1–13%, especially inadvertent detachment is a feared complication when using the Solitaire device and is associated with a poor clinical outcome [5–17]. The literature on inadvertent detachments is sparse, more likely outdated and seems to be an issue of first generation Solitaire devices (AB/FR) since cases of new generation Solitaire devices (2/Platinum/X) have not been reported. Therefore, when confronted with an inadvertent detachment, stentectomy should be the desired strategy in affected patients. Many techniques, ranging from surgical extraction to a variety of different endovascular approaches (balloons, snares, alligator devices, stent-based or combined techniques) to rescue stents have been reported with variable results [10, 12–22].

The purpose of this study was to assess in a porcine model the feasibility and effectiveness of using different stent retrievers in a single retriever technique to rescue detached Solitaire devices.

## Material and Methods

### Animal care

The experiments were approved by the governmental animal protection committee and performed in accordance with the European legislation on the protection of animals (Directive 2010/63/EU) and the National Institute for Health (NIH) guidelines on the care and use of laboratory animals (NIH publication #85-23 Rev. 1985). Experiments were performed in 2 female Swabian Hall pigs (body weight: 40–50 kg) as previously described in detail [23]. The animals had free access to tap water and daily standard food. In order to prevent dehydration a permanent saline infusion was administered. After the experiment the animals were killed with an intracardiac injection of T61 (0.12 mL/kg; MSD Animal Health, Schwabenheim an der Selz, Germany).

### Stent Retrievers

Acquisition of micro-computed tomography (CT) images of the used stent retrievers was performed as previously described in detail [24].

The Solitaire FR (4.0 × 20 mm) (Medtronic) has a tubular design with a longitudinal slit, is closed-cell in design and constructed from laser cut nitinol. The 4‑mm device used has 3 distal markers made of platinum and the 6‑mm device has 4 . Both have one proximal marker made of platinum. Proximally it features an oval, sloping running end [3].

The Trevo ProVue (4.0 × 20 mm; Stryker, Kalamazoo, MI, USA) is a closed tubular and closed-cell design that consists of a flexible tapered nitinol core wire with a tapered distal section. Distally the retriever holds three platinum markers. In addition, platinum wires are integrated into the stent struts [25].

The 3D revascularization device (4.5 × 26 mm; Penumbra, Alameda, CA, USA) is made of nitinol and possesses a partially tubular design with a combination of open and closed cells. It has four chamber-like sections, each with one central marker made of platinum and one additional marker located at the proximal end of the device [26].

The EmboTrap II Revascularization Device (5.0 × 21/33 mm; Cerenovus, Johnson and Johnson, New Brunswick, NJ, USA) comprises a two-layer nitinol structure. The inner structure is tubular designed with a high radial force and provides a flow channel. The outer structure consists of 3‑5 basket-like sections depending on the length of the device. The distal end possesses a closed mesh, which serves as a protection zone. It has four distal gold markers of which one is a tip marker with a length of 4 mm and proximal it has two gold markers [27].

### Intervention

Two neurointerventionalists (W. R. 23 years of interventional experience; A. S. 6 years of interventional experience) performed all the interventions, which were conducted under fluoroscopy by using a monoplane angiographic system (Ziehm Vision imaging, Nuremberg, Germany). The Ultravist 370 (iopromide; Bayer Schering Pharma, Berlin, Germany) was used as a contrast agent. Endovascular procedures were performed after an intravenous bolus injection of 5000 IU heparin (Braun, Melsungen, Germany) and 2 mg nimodipine (Carinopharm GmbH, Elze, Germany).

### Stent Implant Procedure (e.g. Solitaire)

After surgical exposure of the right femoral artery a short 5F sheath was inserted by means of direct puncture. Supported by a 0.035-inch standard angled guide wire (Terumo, Tokyo, Japan) the sheath was replaced by a long 6F Neuron MAX sheath (Penumbra), which was placed in the proximal subclavian artery. Through the sheath a 6F SOFIA (Microvention) intermediate catheter (IC) was inserted. Navigated with a Traxcess 0.014-inch microwire (Microvention), target vessels (axillary arteries and their branches) were reached with either a Rebar-18 or Rebar-27 microcatheter (Medtronic, Irvine, California, USA), depending on the size of the Solitaire device. The microcatheter was then loaded with the detached Solitaire device. Prior to that, the Solitaire device was pulled to the proximal edge of the introducer sheath until the proximal platinum marker was visible. Detachment was manually achieved by bending and twisting the pusher wire bidirectional, representing a type A detachment like previously described [15]. Loading the detached Solitaire device into the microcatheter was achieved by using the stiff-end of a 0.018-inch Muso-Wire (Terumo, Tokyo, Japan). Deployment of the detached Solitaire devices was achieved by gently pushing the Muso-Wire while simultaneously retracting the microcatheter until the Solitaire device was fully unfolded.

**Fig. 1:**
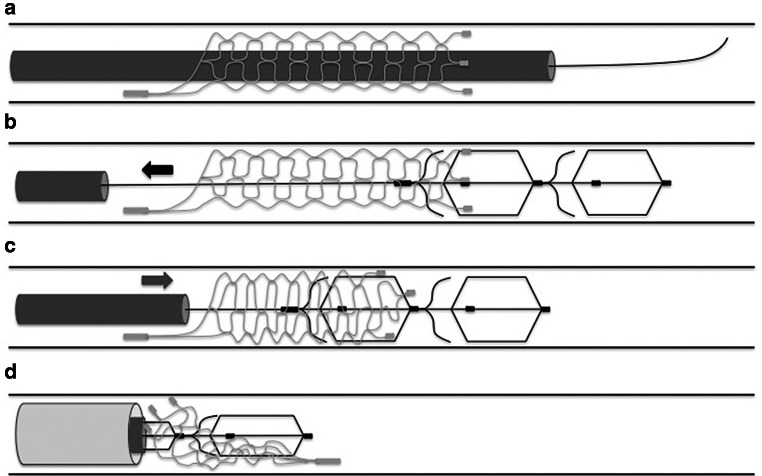
Illustration of the stent-retriever technique with trapping of the detached Solitaire device. First the detached Solitaire device is passed with the microwire and microcatheter (**a**). Then the stent retriever is positioned two thirds distal of the detached Solitaire and one third within the Solitaire (**b**). After that the stent retriever is slowly pulled back until a change in configuration of the Solitaire is noted (**c**). Subsequently, resheathment of the stent retriever is achieved by gently advancing the microcatheter until a mild resistance is felt at the pusher wire of the stent retriever, indicating that the Solitaire is trapped. Following this and under continuous tension of the pusher wire, the complete Solitaire-retriever complex is pulled inside of the IC (**d**)

### Stentectomy Procedure

**Fig. 2:**
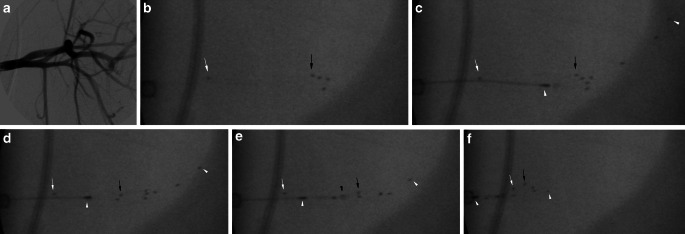
**a** DSA of the left subclavian artery, axillary arteries and its branches. **b** Fluoroscopy image of the detached Solitaire FR 6 × 20 mm. The *white arrow* indicates the proximal marker and the *black arrow* the 4 distal markers. **c** Deployment of the 3D Revascularization Device (*arrowheads* indicate the proximal and distal marker) within the distal two thirds of the Solitaire FR. **d** Retraction of the 3D Revascularization Device with notable change in configuration of the distal markers of the Solitaire FR (*black arrow*). **e** Resheathment of the 3D Revascularization Device by advancing the microcatheter until entrapment of the Solitaire FR (*black arrowhead* indicates the tip of the microcatheter). **f** Partial retraction of the 3D Revascularization Device and trapped Solitaire FR within the IC

Once the Solitaire device was deployed different microcatheters, depending on the stent retriever, were navigated through the Solitaire using a J-shaped Traxcess 0.014-inch microwire (Microvention). The Trevo ProVue was positioned using a Trevo-18 microcatheter (Stryker), the EmboTrap II using a Rebar-18 microcatheter (Medtronic) and the 3D revascularization device using a 0.025-inch Velocity microcatheter (Penumbra). In each maneuver the stent retriever was positioned two thirds distal of the detached Solitaire and one third within the Solitaire so that the distal end of the Solitaire was completely covered. After that the stent retriever was slowly pulled back until a change in configuration of the Solitaire was noted. Subsequently the stent retriever was resheathed by gently advancing the microcatheter until a mild resistance was felt at the pusher wire of the stent retriever, indicating that the Solitaire was trapped. Following this and under continuous tension of the pusher wire, the complete Solitaire-retriever complex was extracted (Fig. 1a–d and Fig. 2a–f). This technique has previously been described to retrieve migrated coils [23, 28, 29].

### Angiographic Evaluation

With each stent retriever 8 detached Solitaire devices at various vessel positions were performed. Rescue was considered successful if the Solitaire device was extracted from the animal. If it was not possible to rescue the Solitaire device, the attempt was declared as having failed. After each rescue maneuver, digital subtraction angiography (DSA) was performed to evaluate vessel complications. The following parameters were assessed:

1. Rescue rates for each clot retriever
2. Rescue time, defined as time between navigation through the Solitaire and successful extraction.
3. Complications: vasospasm, perforation, dissection, entrapment at intermediate catheter, inadvertent deployment and migration of the Solitaire.

### Statistical Analysis

Continuous variables are expressed as means and standard deviations. Continuous variables were tested for normal distribution. Rescue times between the different stent retrievers (TREVO ProVue versus EmboTrap; TREVO ProVue versus 3D Revascularization Device; EmboTrap versus 3D Revascularization Device) were compared by one-way ANOVA followed by post hoc analysis including correction of the α-error according to Bonferroni. Statistical significance was accepted at a two-sided *p* value of <0.05. All data analyses were performed using SPSS Statistics 22 (IBM, Chicago, IL, USA).

## Results

### Vessel and Solitaire Sizes

Using the aforementioned stent retrievers a total of 24 rescue maneuvers were performed in 2 pigs (12 in each pig). With each stent retriever (Trevo ProVue; EmboTrap II; 3D Revascularization Device) a total of 8 rescue maneuvers was performed. For this purpose, the target vessels were the axillary arteries with a mean diameter of 3.38 ± 0.74 mm (animal 1) and 3.29 ± 0.79 mm (animal 2). We implanted a total of 24 Solitaire FR devices of different sizes (4 × 15 mm *n* = 4, 4 × 20 mm *n* = 8, 6 × 20 mm *n* = 4 and 6 × 30 mm *n* = 8).

### Rescue Rate and Time

Successful rescue was achieved in all 24 cases, corresponding to a rescue rate of 100%. The results of the different stent retrievers are listed in detail in Table 1. Using each stent retriever (Trevo ProVue; EmboTrap II; 3D Revascularization Device) we achieved a rescue time of 1–3 min. Comparison of the rescue times of the stent retrievers revealed no statistical significant differences (TREVO ProVue versus EmboTrap, *p* = 0.316; TREVO ProVue versus 3D Revascularization Device, *p* = 0.350; EmboTrap versus 3D Revascularization Device, *p* = 0.122) (Table 1). When pulling the Solitaire-retriever complex inside the IC only a mild resistance was felt. We did not observe a failed rescue. Passage of the Solitaire devices with the microwire and microcatheter was mostly possible at the first attempt using a J-shaped tip on the microwire. The shape of the microwire had to be adjusted in order to achieve the passage only in a few cases.

**Table 1:** Overview of results comparing the applied clot retrievers

Devices	Rescue rate (%)	Time of rescue (s)	Vasospasm (*n*)	Perforation (*n*)	Dissection (*n*)	Entrapment at IC (*n*)	Inadvertent detachment (*n*)	Solitaire migration (*n*)
Trevo ProVue	100	118.85 ± 14.59	0	0	0	0	0	0
EmboTrap II	100	110.14 ± 16.51	0	0	0	0	0	0
3D	100	130.29 ± 27.45	0	0	0	0	0	0

*IC* intermediate catheter

*p* = 0.316 TREVO ProVue versus EmboTrap

*p* = 0.350 TREVO ProVue versus 3D Revascularization Device

*p* = 0.122 EmboTrap versus 3D Revascularization Device

### Complications

We did not observe any cases of vasospasm, perforation or dissection. Neither entrapment of the Solitaire-retriever complex at the intermediate catheter nor migration of the Solitaire devices during navigating was observed.

### Micro-CT of the Rescued Solitaire Devices

**Fig. 3:**
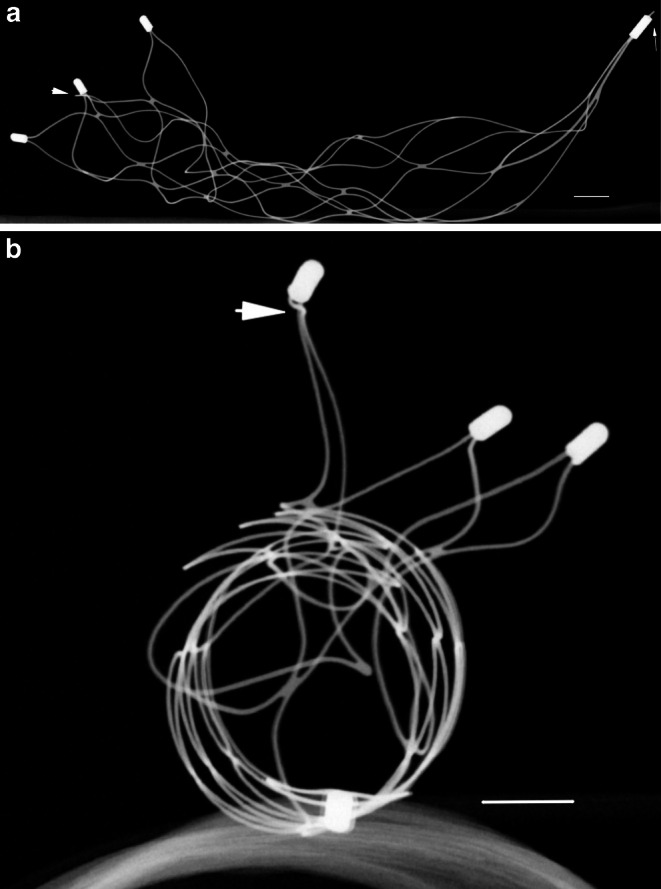
Micro-CT 3D-reconstruction of a rescued Solitaire FR transversal and longitudinal (**a** and **b**), revealing a distinct deformation and invagination. The *thick arrow* indicates one of the trapped distal markers and shows an abnormal bending. The *thin arrow* indicates the detachment zone presenting as a type A detachment. Scale bar = 1 mm

The rescued Solitaire devices were assessed using micro-CT. The images revealed a distinct deformation of each Solitaire device. The point of entrapment was mostly identified directly at one of the distal radiopaque markers, were an abnormal bending of the devices was noted (Fig. 3a). Furthermore, all Solitaire devices were heavily invaginated so that the original open tubular design was no longer recognizable (Fig. 3b).

## Discussion

Inadvertent detachment of the Solitaire device during thrombectomy is a rare complication, but is associated with poor clinical outcome and even increased mortality [15, 16, 30]. Reports in the literature were more frequently published in the early years of mechanical thrombectomy with use of the first generation Solitaire devices (AB/FR) [17, 30]. To date, unexpected detachment of new generation devices (Solitaire 2/Platinum/X) have not been described. Nevertheless, the literature on this topic remains very sparse and most likely outdated. Intracranial arteriosclerotic disease and tortuous vessel anatomy as well as a high number of retrieval attempts seem to contribute to the risk of an unexpected detachment [10, 17]. An in vitro study revealed that detachment most commonly occurs in or around the proximal marker [31]. In a clinical study Castano et al. were able to classify the seen detachments in two types, proximal (type A) and distal (type B) of the proximal marker [15].

To date, data on the management of inadvertent detached Solitaire devices are still limited and no standard protocol has been established. When confronted with such a case, a risk-benefit assessment should be carried out in order to decide between a conservative strategy or a stentectomy. Conservative strategies nowadays include administration of GP IIa/IIIb inhibitors followed by dual antiplatelet therapy and can be potentially catastrophic in ischemic stroke with an increased risk of intracranial hemorrhage [32]. When performing an endovascular stentectomy the extent of neointimal damage to the vessel remains uncertain. Furthermore, complications, such as vessel rupture or dissection must be taken into consideration when using such a procedure.

Many stentectomy techniques have been described in the literature, including the use of balloons [20], snares [19, 21], alligator devices [19] and the merci retriever system [18]. Stent-based techniques using stent retrievers, such as deploy and engage [16] have also been reported and demonstrated promising results. A potential major limitation of the deploy and engage technique might be a weak fixation of the detached stent during rescue [15]. Therefore, another interesting stent-based technique has been described called snare over stent-retriever (SOS) by Chapot et al. and Meyers et al. [22, 33]. With this combined technique the stent-retriever is used to grasp and taper the proximal end of the detached stent so that the snare, which was initially slipped over the microcatheter of the stent-retriever, can be advanced and catch the proximal end of the detached stent. This technique showed promising results with high rescue rates in clinical and experimental reports mainly of stents used for stent-assisted coiling [22, 33].

Detached Solitaire devices were successfully rescued by sole use of a snare as long as the proximal markers were still visualized and not in contact with the vessel wall [15]. Another interesting approach described by Parthasarathy et al. [16] is the loop and snare technique, in which a microwire is looped inside the detached stent and advanced through the mesh back into the parent vessel. With another microcatheter a snare is advanced to catch the microwire so that the stent can be rescued.

In summary rescue maneuvers of Solitaire devices have been described in the literature using sole snares [15], stent retrievers (deploy and engage technique) and loop and snare technique [16].

The aim of the present study was to assess the feasibility and effectiveness of rescuing detached Solitaire devices using different stent retrievers in a porcine model. We chose this animal model because it has already been proven in several studies for the evaluation of endovascular complication management [23, 29]. Despite the very well developed silicone models, they cannot replace the animal model [34]. The applied stent retriever technique has already shown promising results in retrieving migrated coils in many studies using the Trevo ProVue [29] and 3D-Separator [23]. To our knowledge this technique has not been described to rescue detached Solitaire devices. Furthermore, rescue maneuvers using the EmboTrap II Revascularization Device have also not been described in literature.

The results show that overall rescues of detached Solitaire devices of different sizes were successful in 100% (24 of 24 cases). Interestingly, rescue times were not significantly different when comparing the applied stent retrievers. We did not observe any case of entrapment of the Solitaire-clot retriever complex at the intermediate catheter, Solitaire migration, vasospasm, perforation, or dissection.

Presumably the open tubular design of the Solitaire device is most likely the reason for the atraumatic rescue observed in our study. The design allows the Solitaire to be very flexible, which led to an extreme deformation of some devices during the rescue maneuvers. Due to the observed Solitaire deformations we would not recommend this rescue technique for other closed tubular stents.

Taken together, our study reveals that stentectomy of detached Solitaire devices with the applied stent retrievers is a fast and straightforward technique with a manageable risk profile. Compared to the above described techniques using more than one device, the deploy and engage technique or even dedicated rescue devices (snare and alligator), we believe that this technique is simpler, because it is closest to the regular use of a stent retriever in the setting of ischemic stroke. With the growing and more frequent treatment of ischemic stroke using stent retrievers, we believe that neurointerventionalists could benefit from this technique when confronted with an unexpected Solitaire detachment. Thus, we would consider the use of one of the applied stent retrievers as the first choice of therapy in the event of an inadvertent Solitaire detachment.

### Limitations

In the porcine model used, stentectomy of detached Solitaires was performed in vessels representing the sizes of the MCA (M1 and M2 segment), BA and ICA in humans; however, human vessel anatomy is much more challenging in terms of tortuosity. A major limitation was that the detached Solitaire devices were rescued in patent and healthy vessels. In a human setting the stent to be rescued is most likely in an occluded, arteriosclerotically altered vessel with an elongated access path, so navigation is expected to be more challenging than in the animal model used. Furthermore, we were only able to rescue type A detachments of the Solitaire devices, but we do not see any reason why the described technique should not work for type B detachments. Only a small number of rescue maneuvers could be performed for each stent retriever since the supply of Solitaire devices for experimental use was limited.

## Conclusion

This experimental study demonstrates that stentectomy of detached Solitaire devices using stent retrievers is a feasible and effective approach. Rescue rates and rescue times were comparable between the Trevo ProVue, EmboTrap II Revascularization Device and 3D Revascularization Device.

## Abbreviations

BA: Basilar artery
CT: Computertomography
DSA: Digital subtraction angiography
GP: Glycoprotein
IC: Intermediate catheter
ICA: Internal carotid artery
MCA: Middle cerebral artery
